# Supplementary material for: Association of DNA Methylation of the NLRP3 Gene with Changes in Cortical Thickness in Major Depressive Disorder
Source: Int J Mol Sci. 2022 May 21;23(10):5768. doi: 10.3390/ijms23105768 (PMC9143533; doi:10.3390/ijms23105768)
Supplement: Supplementary file 1 [file ijms-23-05768-s001.zip › ijms-1702882-supplementary.pdf]

Table S1. Demographic and clinical characteristics of MDD patients and HCs (total sample).

**Characteristics**	**MDD (n=220)**	**HC (n=82)**	P-value (t, χ^2)
Age	44.55 ± 14.43	31.17 ± 12.31	<0.001 (t = 8.003)
Sex (F/M)	153 / 67	50 / 32	0.158 (χ^2 = 1.991)
Education level			
Elementary and middle school	66	4	
High school or college/university	145	72	<0.001 (χ^2 = 21.502)
Above graduate school	9	6	
HDRS-17 score	17.57 ± 6.68	3.74 ± 5.73	<0.001 (t = 16.587)
Duration of illness (months)	27.86 ± 44.72	NA	NA
Drug-naïve / Medicated patients	82 / 138	NA	NA
Remitted / Non-remitted patients	31 / 189	NA	NA
Medication, n			
SSRI	53		
SNRI	32		
NDRI	7		
NaSSA	14	NA	NA
Other AD	5		
Combination of ADs	27		
AP	28		
Combination of APs	6		

Data are mean ± standard deviation for age, HDRS-17 scores, and illness duration.
P-values for distribution of sex and education level were obtained using a chi-squared test.
P-values for comparisons of age and HDRS scores were obtained using an independent t-test.
Abbreviations: MDD, major depressive disorder; HCs, healthy controls; HDRS-17, 17-item Hamilton Depression Rating Scale; SSRI, selective serotonin reuptake inhibitor; SNRI, serotonin and norepinephrine reuptake inhibitor; NDRI, norepinephrine-dopamine reuptake inhibitor; NaSSA, noradrenergic and specific serotonergic antidepressant; Combination of AD, combinations of two or more types of antidepressants; APs, antipsychotics; ADs, antidepressants; NA, not applicable.
